# Surface Properties of Ultrasonic Vibration-Assisted ELID Grinding ZTA Ceramics

**DOI:** 10.3390/ma15020636

**Published:** 2022-01-15

**Authors:** Zongxia Fu, Fan Chen, Wenbo Bie, Bo Zhao, Xiaobo Wang

**Affiliations:** 1 Henan Province Engineering Research Center of Ultrasonic Technology Application, Pingdingshan University, Pingdingshan 467000, China; fuzongxia@126.com (Z.F.); wenbo187120@163.com (W.B.); 2 School of Mechanical and Power Engineering, Henan Polytechnic University, Jiaozuo 454003, China; zhaob@hpu.edu.cn (B.Z.); wangxb@hpu.edu.cn (X.W.)

**Keywords:** nanocomposite ceramics, residual compressive stress, surface microstructure, surface property, ultrasonic vibration-assisted ELID grinding

## Abstract

This study aimed to explore the evolution of surface properties of nanocomposite ceramics during ultrasonic vibration-assisted electrolytic in-process dressing (UVA-ELID) grinding. First, the trajectory of the grain was analyzed, and the motion was simulated using MATLAB to demonstrate the mechanism of UVA-ELID grinding. The critical grinding depth was also calculated under the effect of ultrasonic vibration. Then, the conventional ELID (C-ELID) and UVA-ELID grinding were compared. The surface properties, including surface residual stress, surface microstructure, surface roughness, and surface morphology, were used to evaluate the effectiveness and feasibility of UVA-ELID grinding. Whether it was conventional C-ELID or UVA-ELID grinding, the residual compressive stress was introduced into the machined surface, while the former was lower than the latter. The microstructure of the UVA-ELID grinding was evenly distributed, and the ductility removal occurred during material removal. The surface roughness of Ra and Rz was reduced by 14.5% and 20.6%, respectively, during the UVA-ELID grinding. The surface morphology was dramatically changed with the help of ultrasonic vibration. In a word, for nanocomposite ceramic, the UVA-ELID grinding can significantly improve surface performance and achieve a better machining effect.

## 1. Introduction

Alumina (Al_2_O_3_) ceramic, as an advanced ceramic, has been widely used in mechanical parts, aerospace, construction, and chemical for its high strength, high hardness, temperature resistance and wear resistance. These properties provide considerable lifetime increases over conventional metal parts [1]. However, the poor impact resistance and toughness restrict the application of Al_2_O_3_ ceramics. One of the most effective methods to overcome this weakness and enhance the toughness of Al_2_O_3_ ceramics is to introduce second-phase particles into the Al_2_O_3_ matrix to form Al_2_O_3_ composite ceramics, for example, adding nano-ZrO_2_ particles [2,3]. On the introduction of these particles into the Al_2_O_3_ matrix, the toughness of Al_2_O_3_ composite ceramics can be significantly enhanced to a certain extent via the phase transition toughening and nano-particle toughening of ZrO_2_ [4,5,6]. Although the performance of Al_2_O_3_ composite ceramics is improved, the mechanical properties are affected by the amount of nano-ZrO_2_ particles and further influence its mechanical properties [7,8]. Compared with metal, nanocomposite ceramics are a hard and brittle material, which is difficult to machine. Recently, many scholars developed high-efficiency and precision machining methods, such as pre-stressed processing, ELID ultra-precision grinding, magnetic polishing technology, ultrasonic-assisted grinding, and ultrasonic vibration-assisted ELID grinding [9]. The ultrasonic vibration-assisted ELID grinding is a novel processing method and also a hotspot across many subjects [10,11].

Kwak et al. [12] combined the ELID with magnetic polishing processing to machine the Zerodure glass-ceramics. They found that the processing efficiency was extremely improved and the surface roughness Ra was 1.7 nm. Nebashi et al. [13] compounded the linear electrode discharge with ELID grinding to obtain high-speed grinding zirconia and silicon nitride ceramics. During processing, the effect of power parameters on grinding force and surface roughness was explored. Kwak et al. [14] investigated the effect of an ultrasonic vibrating table on ELID grinding aluminum nitride ceramics. They found that the material removal rate increased by approximately 36%. Zhao et al. [15] developed the surface roughness prediction model in UVA-ELID grinding nano-composite ceramics and also validated the model. However, the surface properties were not completely explored in the investigation. In addition, the surface properties during UVA-ELID grinding were not examined. The surface properties (surface residual stress, surface microstructure, and surface roughness) play an important role in the evaluation of processing. Consequently, the surface properties under the ultrasonic vibration-assisted ELID grinding need to be investigated.

In this study, the UVA-ELID grinding nanocomposite ceramic was employed to obtain the surface properties. First, the surface formation mechanism during the process was analyzed by establishing the motion equation of grains. The critical grinding depth was obtained under the effect of ultrasonic vibration. Then, the actual grinding experiment was employed to compare C-ELID and UVA-ELID grinding. Finally, the surface properties were investigated during both processing.

## 2. Surface Formation Mechanism of UVA-ELID Grinding

### 2.1. Kinematics Analysis of Grain

**Figure 1:**
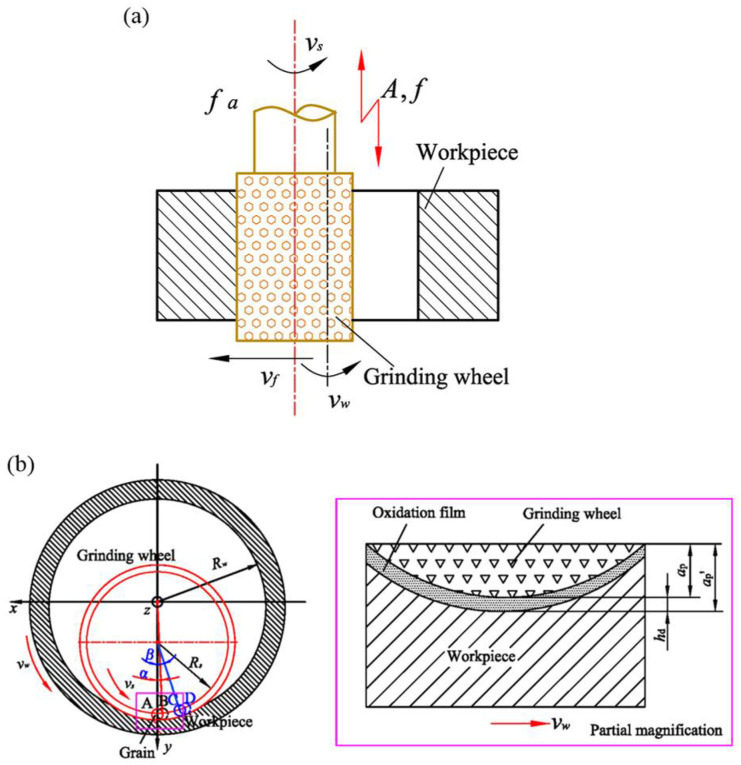
Diagram of UVA-ELID grinding (**a**) Front view (**b**) Top view.

During processing, the trajectory of grain on the workpiece surface determines the characteristics of surface texture [16]. In UVA-ELID grinding, the speed of grinding wheel is $v_{s}$ rotating with the spindle and vibrates along axial direction as shown in Figure 1a. The workpiece rotates with the rotating device, and its speed is $v_{w}$ as shown in Figure 1b.

For the investigation, the following assumptions were made [17]:

(1) The surface of workpiece before and after processing was assumed to an ideal circular curved surface.
(2) The abrasive particles were uniformly distributed on the grinding wheel, and its shape is an ideal spherical shape.
(3) The contour of grains on the grinding wheel remained unchanged during processing due to the online electrolytic dressing of ELID.
(4) The cutting trace of grains was circular, and the circular center of the cutting trace coincided with the center of the workpiece.

According to the geometrical relationship, the trajectory equation of a single grain, A, was obtained under the condition of UVA-ELID grinding.

(1) $$\{\begin{array}{l} x_{A}=(R_{w}+a_{p})\cdot \sin\alpha \\ y_{A}=(R_{w}+a_{p})\cdot (1-\cos\alpha )\\ z_{A}=\frac{R_{w}\cdot \alpha }{v_{s}-v_{w}}\cdot f_{a}+A\cdot \sin(2\cdot \pi \cdot f\cdot \frac{R_{w}\;\cdot \;\alpha }{v_{s}-v_{w}}+\varphi ) \end{array}$$

Similarly, in the Z direction, the trajectory equation of an adjacent grain, B, was as follows.

(2) $$\{\begin{array}{l} x_{B}=(R_{w}+a_{p})\cdot \sin\alpha \\ y_{B}=(R_{w}+a_{p})\cdot (1-\cos\alpha )\\ z_{B}=z_{d}+\frac{R_{w}\cdot \alpha }{v_{s}-v_{w}}\cdot f_{a}+A\cdot \sin(2\cdot \pi \cdot f\cdot \frac{R_{w}\;\cdot \;\alpha }{v_{s}-v_{w}}+\varphi ) \end{array}$$

where *R_w_* is the inner radius of the workpiece, *a_p_* is the grinding depth, *z_d_* is the distance between grain B and abrasive A in the Z direction, *α* is the rotation angle of the grinding wheel, *f_a_* is the feed speed of the grinding wheel along the axial direction, *f* is the ultrasonic frequency, *A* is the ultrasonic amplitude, and *φ* is the initial phase angle.

In the peripheral direction of the grinding wheel, the trajectory equations of the grinding grains C and D and adjacent grains A and B were as follows:

(3) $$\{\begin{array}{l} x_{C}=(R_{w}+a_{p})\cdot \sin\alpha +R_{s}\cdot \sin\beta \\ y_{C}=(R_{w}+a_{p})\cdot (1-\cos\alpha )+R_{s}\cdot \cos\beta \\ z_{C}=\frac{R_{w}\cdot \alpha }{v_{s}-v_{w}}\cdot f_{a}+A\cdot \sin(2\cdot \pi \cdot f\cdot \frac{R_{w}\;\cdot \;\alpha }{v_{s}-v_{w}}+\varphi ) \end{array}$$

(4) $$\{\begin{array}{l} x_{D}=(R_{w}+a_{p})\cdot \sin\alpha +R_{s}\cdot \sin\beta \\ y_{D}=(R_{w}+a_{p})\cdot (1-\cos\alpha )+R_{s}\cdot \cos\beta \\ z_{D}=z_{d}+\frac{R_{w}\;\cdot \;\alpha }{v_{s}-v_{w}}\cdot f_{a}+A\cdot \sin(2\cdot \pi \cdot f\cdot \frac{R_{w}\cdot \alpha }{v_{s}-v_{w}}+\varphi ) \end{array}$$

where *R_s_* is the inner radius of the grinding wheel, *β* is the rotation angle of grain C from grain A in the peripheral direction with the center of the grinding wheel.

**Figure 2:**
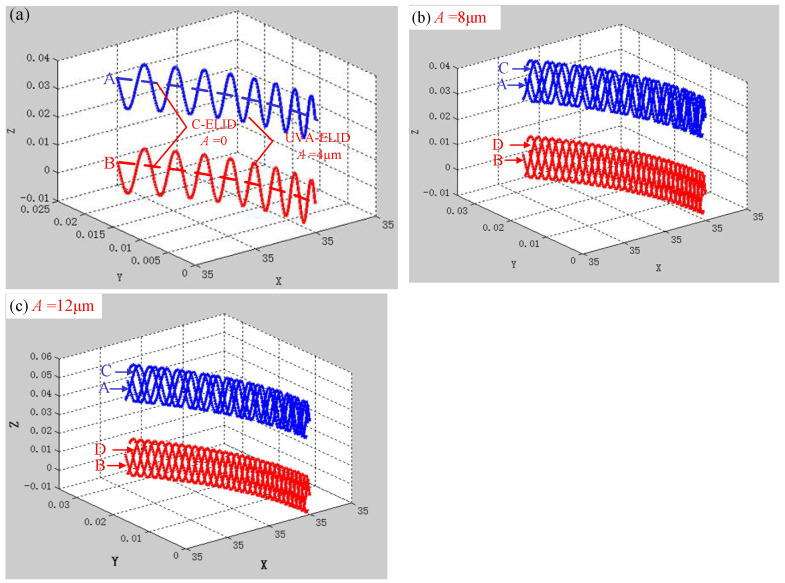
Simulation trajectory of grains in both processing. (**a**) C-ELID *A* = 0 and UVA-ELID *A* = 4 μm; (**b**) UVA-ELID *A* = 8 μm; (**c**) UVA-ELID *A* = 12 μm.

According to the aforementioned trajectory equations, the trajectory in Figure 2 during both processing could be obtained using MATLAB. As shown in Figure 2a, the trajectory of grain in C-ELID was an arc curve, while in the UVA-ELID, the sinusoidal trajectory was presented under the action of ultrasonic vibration. Figure 2b,c show that the trajectory of multiple grains was overlapped in the contact area, and the alternating overlap became more obvious with the increase in ultrasonic amplitude. In other words, the greater ultrasonic amplitude was beneficial to be interfered with each grains trajectory and generated the fishing-like mesh, which made the surface texture more dense and uniform, and presented the reticulate microstructure.

### 2.2. Critical Grinding Depth

During the grinding processing, the action of grains on the workpiece was often regarded as an indentation experiment. It was assumed that the workpiece should be a rigid-plastic. According to Swain and Lawn [18], the relationship between the contact force *P_L_* and the feature size *a* was expressed as follows.

(5) $$P_{L}=\xi \cdot H_{v}\cdot a^{2}$$

where *ξ* is the geometrical factor of the diamond indenter, *H_v_* is the hardness of the workpiece.

**Figure 3:**
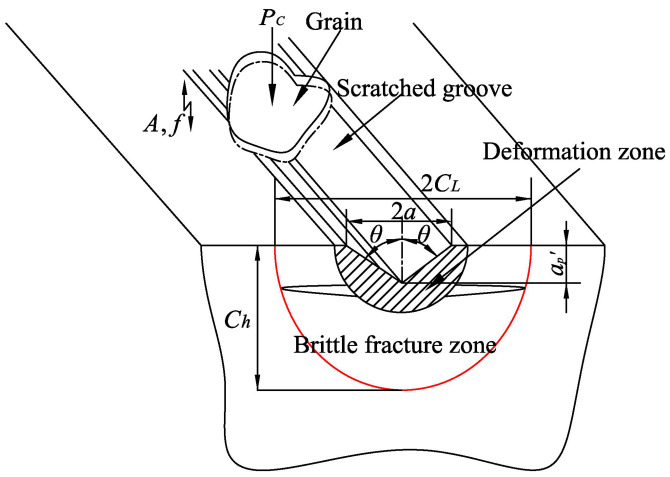
Material removal of a single grain under UVA-ELID grinding.

As shown in Figure 3, when the applied force was less than the critical force *P_c_*, the plastic deformation controlled the material removal. In contrast, the material was removed by crack propagation. Wilshaw et al. [19] believed that the critical force *P_c_* could cause crack propagation and was expressed as:

(6) $$P_{c}=\lambda_{0}K_{IC}{\left(K_{IC}/H_{v}\right)}^{3}$$

Figure 3 shows that the feature size *a* was relative to the grinding depth *a*_p_’ and the half-angle of indenter or grain θ, namely, $a=a_{p}\prime \tan\theta$.

Equation (5) could be written as follows.

(7) $$P_{L}=\frac{1}{2}\cdot \xi \cdot H_{v}\cdot a_{p}\prime^{2}\tan^{2}\theta$$

Combining Equations (6) and (7), the critical indentation depth or the critical ductile depth of a single grain could be obtained.

(8) $$a_{p}\prime =\frac{{K_{IC}}^{2}}{{H_{v}}^{2}\tan\theta }\sqrt{\frac{2\lambda_{0}}{\xi }}$$

Equation (8) was obtained in the static condition, and it was not used to reflect dynamic grinding. A correction factor *k_u_* was introduced to Equation (8) to reflect the effect of ultrasonic vibration. The critical ductile depth under the action of ultrasonic vibration could be obtained as follows:

(9) $$a_{p}\prime =k_{u}\cdot \frac{{K_{IC}}^{2}}{{H_{v}}^{2}\tan\theta }\sqrt{\frac{2\lambda_{0}}{\xi }}$$

As shown in Figure 2b, the grinding depth during ELID could be obtained as:

(10) $$a_{p}\prime =a_{p}+h_{d}$$

where *a_p_* is the actual grinding depth, and *h_d_* is the depth of dissolution on the wheel due to the effect of electrolysis.

According to Faraday’s law, the following relationship could be determined:

(11) $$\frac{dV_{v}}{dt}=\eta \frac{M\cdot I}{z\cdot F\cdot \rho }$$

where *V_v_* is the volume metal-bonded material removed, *t* is effective electrolytic time, *η* is the current efficiency, *M* denotes the molecular weight of metal-bonded, *z* is the valence of the metal, *ρ* is the density of metal-bonded, and *F* is the Faraday constant.

The depth of dissolution on the wheel *h_d_* could be expressed as follows:

(12) $$h_{d}=\frac{V_{V}}{A_{a}}=\eta \frac{M\cdot I\cdot D\cdot t}{z\cdot F\cdot \rho \cdot A_{a}}$$

where *A_a_* is the effective area of anode for conduction.

According to Ohm’s law, the current *I* could be defined as:

(13) $$I=\frac{U}{R}$$

During the ELID grinding, the total resistance *R* includes the electrolyte resistance *R_e_* and the oxidation film resistance *R_f_* as shown Equation (14):

(14) $$R=R_{e}+R_{f}=\frac{\rho_{e}h_{e}}{A_{e}}+\frac{\rho_{f}h}{A_{e}}$$

where *ρ_e_* is electrolyte resistivity, *h_e_* is inter-electrode clearance, *ρ*_0_ is oxidation film resistivity, and *A_e_* is the effective area of the cathode.

Substituting Equation (14) into Equation (13), the current *I* could be obtained as:

(15) $$I=\frac{U}{R}=\frac{UA_{e}}{\rho_{e}h_{e}+\rho_{f}h}$$

The *h_d_* could be expressed as follows:

(16) $$h_{d}=\eta \frac{M\cdot I\cdot D\cdot t_{a}}{z\cdot F\cdot \rho \cdot A_{a}}=\frac{\eta \cdot M\cdot D\cdot t\cdot U\cdot A_{c}}{z\cdot F\cdot \rho \cdot A_{a}(\rho_{e}\cdot h_{e}+\rho_{f}\cdot h)}$$

Combining Equations (9), (10), and (16), the actual grinding depth $a_{p}$ could be obtained as follows:

(17) $$a_{p}=k_{u}\cdot \frac{{K_{IC}}^{2}}{{H_{v}}^{2}\cdot \tan\theta }\sqrt{\frac{2\lambda_{0}}{\xi }}-\frac{\eta \cdot M\cdot D\cdot t\cdot U\cdot A_{e}}{z\cdot F\cdot \rho \cdot A_{a}(\rho_{e}\cdot h_{e}+\rho_{f}\cdot h)}$$

It was observed from Equation (17) that the critical grinding depth was composed of two parts. The first part was mainly determined by the inherent characteristic of the material and the ultrasonic parameters (ultrasonic frequency and amplitude). The second part was determined by the electrical parameters of ELID grinding. During the UVA-ELID, the critical grinding depth was larger than that in the C-ELID. According to Equation (17), the critical grinding depth was approximately 3.73 μm using MATLAB. Therefore, the ductile-regime removal of the material was improved under the action of ultrasonic vibration.

## 3. Experimental Setup and Methodology

### 3.1. Experimental Setup

**Figure 4:**
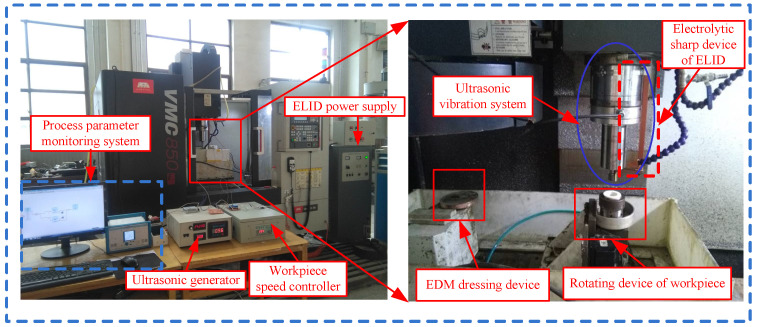
Experimental setup of UVA-ELID grinding.

The C-ELID and UVA-ELID grinding tests were performed on a three-axial machining center (VMC850E, SMTCL, Shenyang, China). As shown in Figure 4, the experimental setup included the ELID power supply, electron discharge machining (EDM) dressing device, ultrasonic vibration system, the sharp electrolytic device, and processing parameter monitoring system. The ELID power supply was mainly used to provide the energy for the processing and regulate electrical parameters. The EDM dressing device was fixed on the left side of the worktable for dressing the grinding wheel. The electrolytic sharp device and the ultrasonic vibration system were fixed on the spindle using a clamp. 

### 3.2. Experimental Condition 

The ZTA ceramics with a zirconia content of 20% was used to prefabricate into a ring. The experimental conditions are shown in Table 1. During the test, the C-ELID grinding and UVA-ELID grinding were conducted by switching on or off the ultrasonic generator.

### 3.3. Surface Property Test

The workpiece was cleaned with hydrochloric acid and kept dry before tested. The residual stress in the surface was measured with the help of the PROTO X-ray by using the XRD method. The surface texture was observed under an optical microscope (VHX-2000, KEYENCE, Osaka, Japan) with higher resolution. The three-dimensional white interferometer (Talysurf CCI6000, Taylor Hobson, Leicester, UK) was mainly utilized to measure the 3D surface topography and obtain correspond surface roughness of machined surface. The surface morphology was observed through the scanning probe microscope (CSPM-2000, Being Nano-Instruments Co., Ltd., Guangzhou, China). Three points were gauged for each set of parameters to gain reliable data, and the average value was taken as the final result.

## 4. Results and Discussions

### 4.1. Surface Residual Stress

**Figure 5:**
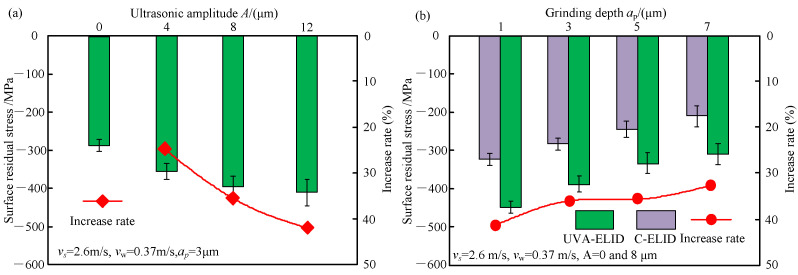
Effect of parameters on the surface residual stress (**a**) Ultrasonic amplitude (**b**) Grinding depth.

Figure 5 presents the effect of parameters on the surface residual stress of the nanocomposite ceramic under C-ELID and UVA-ELID grinding. As shown in Figure 5, the residual compressive stress was introduced through the both processing. Figure 5a shows that the residual compressive stress increased by 42.1% with the increase in ultrasonic amplitude. It was mainly attributed to the fact that the ZTA belongs to the nanocomposite ceramics with phase transition. During the processing, when the grinding stress attained the critical stress of phase transition, the volume expanded by 3–5% owing to the nanometer zirconia particles transition from the metastable tetragonal phase to the monoclinic phase. It led to the generation of higher residual compressive stress by the transformation layer. In addition, the residual compressive stress during the UVA-ELID grinding was higher than that during the C-ELID. The higher ultrasonic amplitude exhibited a strong impact on the workpiece and caused the phase transition to a certain extent. In addition, it resulted in the flank face of grains reciprocating ironing on the surface. On the contrary, it decreased with an increase in grinding depth in Figure 5b. The residual compressive stress was enhanced by 41.3% during the UVA-ELID grinding compared with the C-ELID grinding. This was mainly attributed to the fact that the surface residual stress was the coupled action of material phase transition, hot plastic deformation caused by grinding heat, and cold plastic deformation caused by mechanical stress. When the grinding depth was smaller than the critical grinding depth, the grinding temperature was relative lower. With the increase in grinding depth, the grinding force increased and the friction between the grain and workpiece was enhanced, which resulted in an increase in the grinding temperature. The higher grinding temperature would generate tensile stress on the surface and offset some of the compressive stress. Therefore, the surface compressive stress value presented a declining tendency. In C-ELID grinding, the decline rate was approximately 10% with the grinding depth 1–5 μm, while it was approximately 20% with the grinding depth larger than 5 μm. However, for the UVA-ELID grinding, the descending rate reached 20% with the grinding depth 1–3 μm, and then remained at approximately 10%. The difference between both processing indicated that the overlapping trajectory of grains led to a decline in the grinding temperature under the action of ultrasonic vibration, and the instantaneous impact could promoted the martensite phase transition to generate higher residual stress. Therefore, the decline rate with the increase in grinding depth during UVA-ELID was not evident compared with the C-ELID grinding when the grinding depth exceeded the critical depth.

### 4.2. Surface Roughness

**Figure 6:**
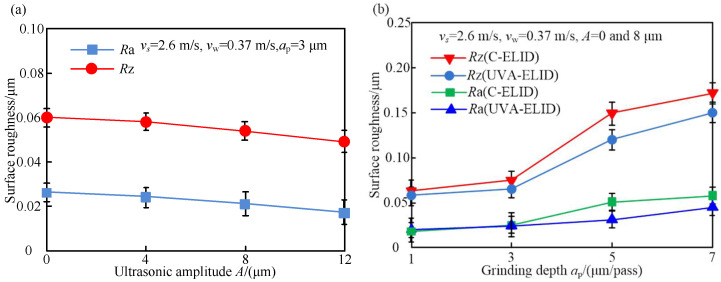
Effect of parameters on the surface roughness (**a**) Ultrasonic amplitude (**b**) Grinding depth.

The effect of parameters on the surface roughness during both processing is shown in Figure 6. As can be seen from Figure 6a,b, the surface roughness (*R*a and *R*z) decreased with the increase in ultrasonic amplitude but increased with the grinding depth. It was attributed to the fact that the increase in ultrasonic amplitude strengthened the interference between the grain trajectories and led to more material removal. The surface roughness *R*a and *R*z decreased by 14.5% and 20.6%, respectively, with grinding depth during the UVA-ELID compared with the C-ELID grinding. The surface roughness was enhanced obviously with the grinding depth in the range of 3–5 μm, including the critical grinding depth, while it displayed a linear increase when the grinding depth was below this range. This indicated that the material removal changed within the scope of grinding depth. When the grinding depth exceeded this range, the variation in roughness was not obvious. This was the reason that the higher instantaneous impact under ultrasonic vibration acted on the surface and led to a dramatic change in micro-topography. Compared with the instantaneous impact force, the average acting force on the surface was smaller. It resulted in a decrease in surface roughness (*R*a), and the quality was improved. 

**Figure 7:**
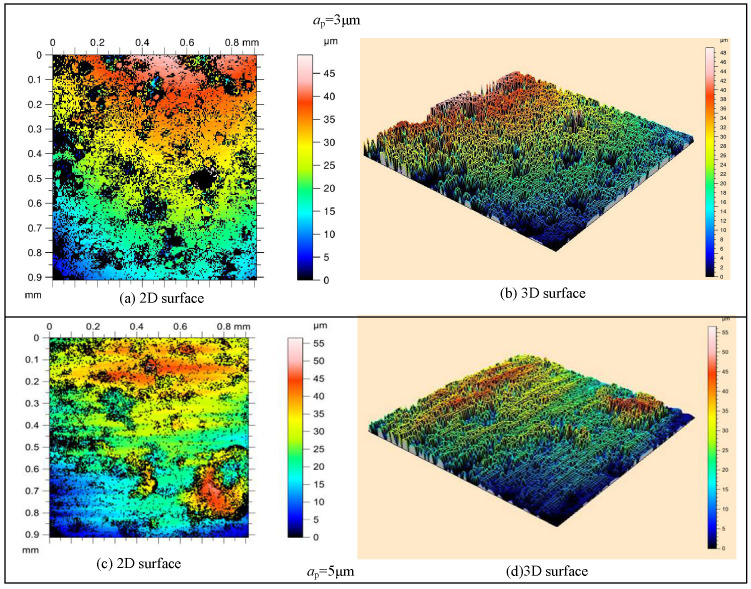
Surface microstructure during C-ELID observed using a white interferometer.

**Figure 8:**
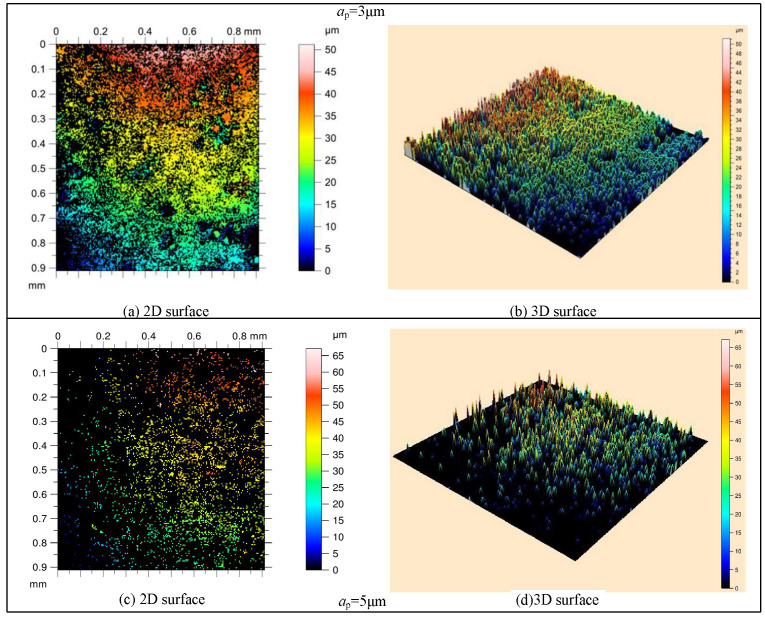
Surface microstructure during UVA-ELID observed using a white interferometer.

The surface microstructures obtained through C-ELID and UVA-ELID are shown in Figure 7 and Figure 8. First, the surface microstructure observed from the optical microscope was more uniform, and the material removal presented ductile removal in UVA-ELID grinding compared with C-ELID grinding. It deteriorated with the increase in grinding depth during the C-ELID grinding. As the grinding depth was 5 μm exceeding the critical depth, the microstructure could not be generated on the surface, while its spacing and height enhanced during UVA-ELID grinding. Simultaneously, the reticulate microstructure was formed on the surface, which was in good agreement with the grain trajectory under the action of ultrasonic vibration. It was attributed to the fact that the contour of grains was better during the ELID with the help of ultrasonic vibration. The effect of ultrasonic vibration changed the distribution of grains on the grinding wheel, and caused its height to remain relatively uniform. In addition, a layer of oxide film generated under the ELID electrolysis also participated in polishing and grinding [17]. During the UVA-ELID grinding, the high-frequency vibration would clean up the debris under the oxide film in time and reduce its scratch on the surface. Therefore, better surface quality was obtained during the UVA-ELID grinding.

### 4.3. Surface Morphology

**Figure 9:**
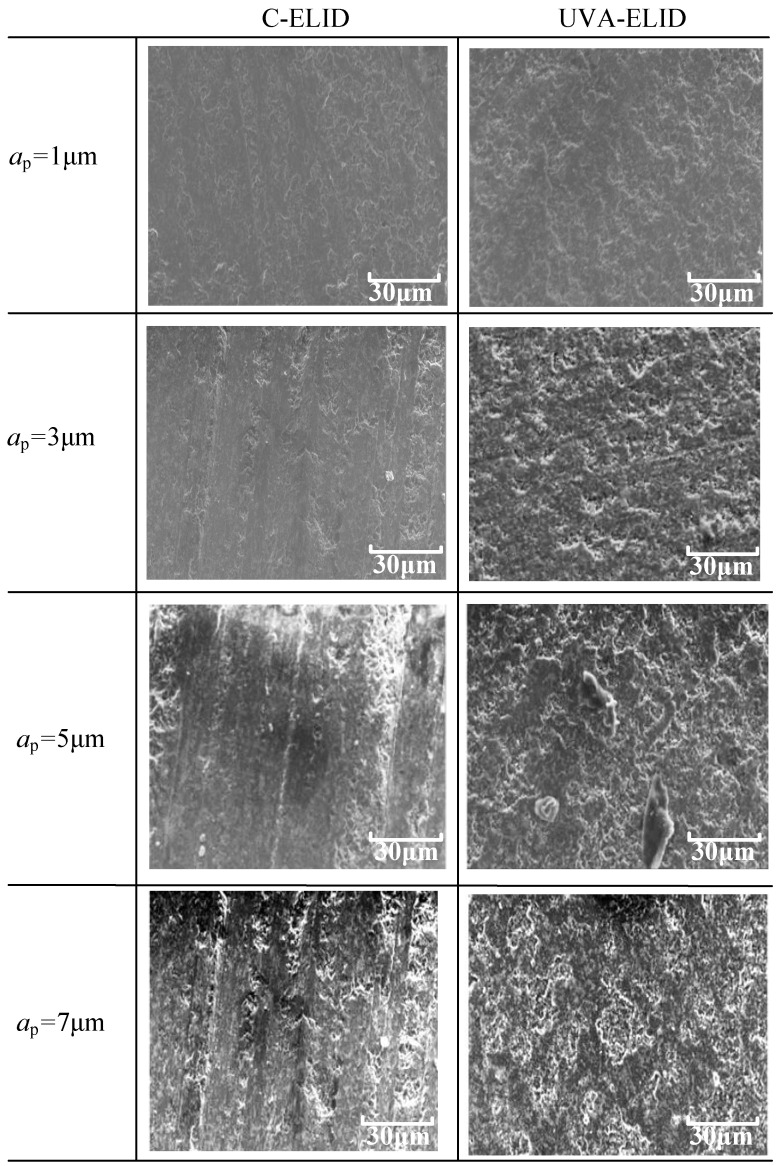
Surface morphology of the workpiece under different processing.

The surface morphology under different processing parameters is presented in Figure 9. The grooves on the surface of C-ELID were obviously presented on the machined surface when the grinding depth was less than 3 μm. It demonstrated that the materials were mainly removed by plastic deformation. As shown in Figure 9, when the grinding depth was 5 μm, the surface quality gradually deteriorated with the appearance of plowing ridges. With an increase in grinding depth, the plowing ridges were obvious on the surface, with some fractures. In contrast, the UVA-ELID could change the mode of material removal; even at the larger grinding depth, the ductile remove took place, which improved the surface quality to a certain extent. It was attributed to the fact that the effect ultrasonic vibration could change the characteristic of the nanocomposite ceramic. When the grain impacted the surface with a higher ultrasonic amplitude, the microhardness of the workpiece changed. That is to say, the internal vibration stress generated from ultrasonic vibration offset some of the existing internal stress, which was also called equivalent hardness. The large internal vibration stress led to an obvious decrease in equivalent hardness and softened the surface layer [20]. Therefore, the softening effect of ultrasonic vibration during UVA-ELID prompted material removal and achieved a higher quality.

## 5. Conclusions

In this study, the surface properties were investigated based on the mechanism of surface formation during UVA-ELID grinding. Based on the findings, the following conclusions were drawn.

(1) The trajectory of grains during the UVA-ELID displayed sinusoidal movement. The overlapping trajectory led to the surface microstructure more dense and uniform, presenting a reticulate microstructure. It was beneficial to reducing the surface roughness and improving the surface properties. In addition, the critical grinding depth during the UVA-ELID grinding was improved and the material removal was also changed.
(2) The residual compressive stress was introduced into the surface during both processing. Under the action of ultrasonic vibration, the residual compressive stress increased by 42.1% compared with that during the C-ELID. During the UVA-ELID grinding, the high-frequency vibration could generate the reticulate microstructure on the surface. In addition, the surface roughness *R*a and *R*z decreased by 14.5% by 20.6%, respectively.
(3) The characteristic of the nanocomposite ceramic changed during the UVA-ELID grinding, and the plastic removal occurred at the lower grinding depth. Therefore, the surface morphology was better with an increase in grinding depth.

## Figures and Tables

**Table 1:** Experimental conditions.

Types	Parameters	Value
Trimming	Voltage (V)	120
	Wheel speed (r/min)	1000
Workpiece	Outer diameter (mm)	60
	Inside diameter (mm)	35
	Height (mm)	40
Grinding parameters	Wheel speed (m/s)	2.6
	Grinding depth (μm/pass)	1, 3, 5, 7
	Workpiece speed(m/s)	0.37
Ultrasonic parameters	Frequency (kHz)	25.3
	Amplitude (μm)	0, 4, 8, 12

## Data Availability

All data generated or analyzed during this study are included in the present article.
